# Evaluation of Breast Skin/Nipple-Areolar Complex Sensation and Quality of Life after Nipple-Sparing Mastectomy Followed by Reconstruction

**DOI:** 10.3390/medicina60101655

**Published:** 2024-10-09

**Authors:** Beatriz Soares Domingues Polita, Jānis Lapinš, Ansis Ģīlis, Michal Grucki, Arvids Irmejs, Jānis Gardovskis, Jeļena Maksimenko

**Affiliations:** 1 Department of Surgery, Rīga Stradiņš University, LV-1007 Rīga, Latviajanis.gardovskis@stradini.lv (J.G.); 2 Pauls Stradiņš Clinical University Hospital, LV-1002 Rīga, Latvia; 3 Institute of Oncology and Molecular Genetics, Rīga Stradiņš University, LV-1007 Rīga, Latvia

**Keywords:** nipple-sparing mastectomy, breast reconstruction, sensation preservation, quality of life, BREAST-Q

## Abstract

*Background and Objectives*: Sensation of the breast skin and nipple-areolar complex (NAC) is commonly assumed to be diminished or completely absent following nipple-sparing mastectomy (NSM) with implant- or expander-based reconstruction. The purpose of this cohort study was to evaluate breast skin and NAC long-term touch pressure sensibility, from 1 month to 1 year, after NSM followed by reconstruction with an implant or expander, and patient quality of life (QoL), hypothesizing that sensibility may diminish with a small progressive return throughout the postoperative period. *Materials and Methods*: This was achieved by performing sensation tests using Semmes-Weinstein monofilaments (SWM) in nine predefined points of the breast and NAC, a two-point discrimination test (TPD) in the four quadrants of the breast, and QoL assessment using the BREAST-Q. We evaluated 42 patients in Pauls Stradiņš Clinical University Hospital, with a total of 66 breasts, who underwent NSM between 2021 and 2023, performing the breast sensation tests before surgery and postoperatively at 1/3/6 months and 1 year. The BREAST-Q was administered to assess patient satisfaction and well-being. *Results*: Our results reflect a decline in breast skin and NAC sensation in the 1-month evaluation after NSM (mean: 4.67) when compared to the assessment before surgery (mean: 2.57), with a small progressive return reflected in the 3 months (mean: 3.79), 6 months (mean: 3.68), and 1-year evaluations (mean: 3.14). The following were the mean scores obtained from the BREAST-Q: Psychosocial Well-being (mean: 66), Sexual Well-being (mean: 50), Satisfaction with Breasts Pre-OP (mean: 58), satisfaction with breast reconstruction (mean: 52), Satisfaction with Implants, Satisfaction with nipple reconstruction, Physical Well-being Chest, Adverse effects of radiation, and Satisfaction with Information. *Conclusions*: This study confirms that sensibility diminishes after this procedure, as observed when comparing the sensation evaluation results before the operation with the 1-month evaluation, reflecting a small progressive return in the following months.

## 1. Introduction

Sensation of the breast skin and nipple-areolar complex (NAC) is commonly assumed to be diminished or completely absent following nipple-sparing mastectomy (NSM) with implant- or expander-based reconstruction. The impact of sensation loss on the physical, psychosocial, and sexual well-being of patients has been discussed in the current literature, highlighting the importance of considering the long-term effects of breast skin and NAC sensation after NSM with reconstruction to optimize patients’ QoL results during the postoperative period. Research suggests that patients with better breast skin and NAC sensation preservation following this procedure will present stronger quality of life (QoL) results [1]. Pursuing this further, despite describing a decrease in NAC sensation when compared to preoperative examinations, most patients report having measurable retained sensation in the NAC after NSM, with most of the women reporting preserved sensation in at least one NAC [2].

Concerning the matter in question, experts have developed and validated a tool to measure overall well-being, satisfaction, and QoL in women who have undergone NSM with reconstruction. They found that the BREAST-Q is a reliable instrument for measuring such outcomes in women who have undergone this surgery and that it can provide important information for clinical care planning [3,4]. Furthermore, it is important to consider the outcomes and complications of NSM followed by breast reconstruction. This procedure is an oncologically safe and effective technique with high rates of patient satisfaction, natural-appearing cosmetic results, low complication rates, and low incidence of occult disease. In light of such compelling findings, NSM with implant-based reconstruction (IBR) is becoming the preferred approach to breast reconstruction [5,6,7].

The purpose of this study was to evaluate long-term breast skin and NAC sensation in patients who have undergone NSM followed by reconstruction with an implant or expander, as well as their QoL. To best interpret the progression of sensibility return, we performed sensation tests before surgery as well as postoperatively at 1/3/6 months and 1 year. Using Semmes-Weinstein monofilaments, we were able to assess touch pressure sensibility in our patients. It is a non-invasive evaluation using varying-size monofilaments, corresponding to different pressure sensibility thresholds, in nine predefined points/areas of the breast and NAC. This assessment, coupled with the two-point discrimination test, provides valuable information on the impact of the procedure on patient sensory perception at these predefined points. These findings allow us to draw conclusions regarding the evolution of sensation values during the postoperative follow-up period. Beyond sensory assessment, we administered the BREAST-Q questionnaire to measure the influence of this procedure on patients’ QoL. This involved the evaluation of several domains including overall patient satisfaction, quality of life, and satisfaction with reconstruction results. Understanding the effects of this procedure on patients’ QoL is imperative to provide a high standard of care.

The conclusions obtained from the sensibility evaluation, which hypothesized that breast skin and NAC sensation may diminish after the procedure with a small progressive return during the postoperative period, combined with the patient-reported outcome measures (PROM) questionnaire, will contribute to a better understanding of the significance of preserving breast skin and NAC sensation throughout the postoperative process, thereby optimizing prospects for improving patients’ QoL outcomes. Extended follow-up periods and the use of alternate surgical techniques, such as nerve transfers and nerve coaptation, should be built upon in future studies to investigate the implications of long-term sensory function and monitor patient health.

## 2. Materials and Methods

### 2.1. Patient Eligibility

We evaluated 42 patients at Pauls Stradiņš Clinical University Hospital who underwent NSM with reconstruction between 2021 and 2023, with a total of 66 breasts meeting the inclusion criteria. The characteristics of the research population were established according to specific inclusion and exclusion criteria that fit our research goals, and participants were recruited from among patients referred for NSM with reconstruction.

The inclusion criteria included patients who had undergone nipple-sparing mastectomy with immediate reconstruction with an implant or expander, whose nipple was not excised, who were able to provide written consent, who were able to answer the questionnaire, and who were over the age of 18 years.

Exclusion criteria applied to patients for whom sensibility examination could not be performed, a different surgical technique other than nipple-sparing mastectomy was adopted, the nipple was excised, those who were not able to cooperate in the examination/questionnaire, and those who did not meet the inclusion criteria.

### 2.2. Data Collection and Statistical Methodology

Essential patient data were collected, including clinical information (age, type of surgery, reconstruction details, incision site, and therapy received), the BREAST-Q questionnaire in paper format, and sensory assessment data. Subsequently, all data were transferred to an electronic format in Microsoft Excel (version 16.89.1) and IBM SPSS Statistics version 29.0.0.0 (241) released in 2022 for statistical data analysis, with confidentiality maintained through patient identification numbers.

Observed data were characterized using statistical descriptors, namely mean values, maximum and minimum scores, and counts expressed as percentages. The BREAST-Q raw scores were converted into domain Rasch-Transformed (RT) scores, which were scaled from 0 to 100. Using a paired-sample *t*-test, we compared the obtained results, using the preoperative measurements as the control group, to observe which areas of the breast skin and NAC had the best-preserved sensitivity. Further parameters were evaluated to check for statistically significant effects. The results were presented with 95% confidence intervals, and the significance threshold was defined as *p* < 0.05.

For a better understanding of the differences in mean sensation values at the various time points, we performed an ANOVA (Analysis of Variance) test to determine if there were statistically significant differences present. The ANOVA test resulted in a *p*-value <0.001. This *p*-value is significantly below the common alpha level of 0.05, indicating that there are significant differences in the mean sensation values across the different time points for each of the 9 points on the breast. This suggests that the sensation values do not remain constant over the study period, supporting the hypothesis that sensation changes significantly after surgery. Furthermore, a paired *t*-test was performed comparing the sensation values at the preoperative time point with the 12-month evaluation. For points 1, 2, 5, 6, and 9, the *p*-values were less than 0.05, indicating that there is a statistically significant difference in sensation values between these time frames. However, points 3, 4, 7, and 8 show a *p*-value greater than 0.05, implying that there is no statistically significant difference and that the sensation values at these specific points are similar at both time periods. This suggests that one year after surgery, these points return to similar preoperative values and show greater sensation recovery. These results imply that the medial side of the breast has greater recovery or maintenance of sensation function compared to the lateral side, likely due to damage of the LCBs of the 2nd to 6th intercostal nerves during surgery. Finally, a power analysis was conducted using G*Power 3.1.9.6. With a sample size of 42 patients (66 breasts measured at five different time points), with an alpha level of 0.05 and a partial eta squared effect size (η^2^p) of 0.844, the power was calculated to be 0.98.

Delayed or immediate reconstruction, incision sites, axillary surgery, implant or expander, chemotherapy, and age were the evaluated parameters to check for statistically significant effects.

### 2.3. Sensory Testing Measurements and Administration

**Figure 1:**
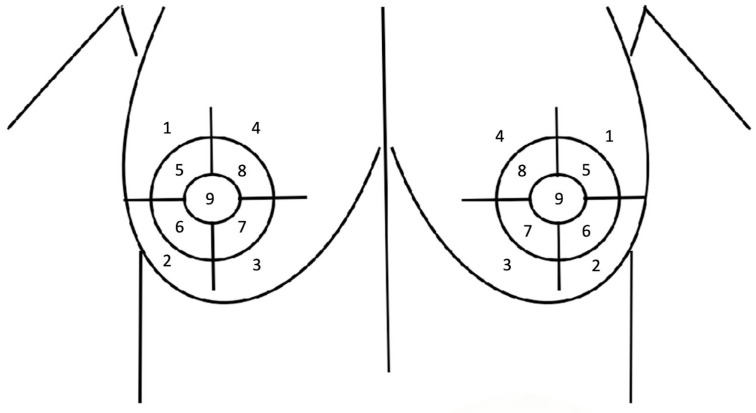
Nine predefined points for sensation evaluation of the breast skin and NAC.

Sensory tests were performed to measure sensation in the breast skin and NAC before surgery, as well as postoperatively at 1/3/6 months and 1 year. The sensitivity pressure threshold was evaluated with Semmes-Weinstein monofilaments using monofilaments of varying sizes, corresponding to different pressure sensibility thresholds, in nine predefined points/areas of the breast and nipple-areolar complex. Points 1–4 were measured 3 cm from the areola margin at 12, 3, 6, and 9 o’clock, and represented the area of the breast skin to be measured. Points 5–8 were measured 1 cm from the nipple margin at 12, 3, 6, and 9 o’clock and represented the areas of the areola to be measured. Point 9 was determined as the nipple (Figure 1, Table 1).

After the test was performed, the recorded values were attributed to five different categories. Green monofilaments represent normal touch, blue diminished light touch, purple diminished protective sensation, red loss of protective sensation, or only deep pressure sensation (Table 2).

A two-point discrimination test was performed in the four quadrants of the breast, and the results described whether the patient was able to perceive two points, only one point, or no point at all. These results could be further interpreted into five categories: normal, fair, poor, protective sensation, and anesthesia (Table 3).

### 2.4. BREAST-Q Administration

Experts have developed and validated a tool to measure overall well-being, satisfaction, and quality of life in women who have undergone nipple-sparing mastectomy with reconstruction. They found that the BREAST-Q is a reliable instrument for measuring such outcomes in women who underwent this surgery and that it can provide important information for clinical care [3,4]. The BREAST-Q questionnaire was used to assess patients’ quality of life in the psychosocial and physical domains. This included overall patient satisfaction, QoL, and satisfaction with reconstruction results. The BREAST-Q raw scores were converted into domain Rasch-Transformed (RT) scores, which were scaled from 0 to 100. Higher scores indicate better health-related quality of life outcomes and higher satisfaction.

## 3. Results

### 3.1. Evaluation of Pressure Sensitivity in the Breast Skin and Nipple-Areolar Complex

We evaluated 42 patients at Pauls Stradiņš Clinical University Hospital, who underwent NSM with reconstruction between 2021 and 2023, with a total of 66 breasts meeting the inclusion criteria. The average age of our patients was 45 years (range: 28–63) years. From this pool of patients, 24 patients underwent bilateral NSM, and 18 had unilateral NSM or the counter breast did not meet the inclusion criteria to correspond to the intended objectives of this study. Regarding reconstruction techniques, 22 patients received direct-to-implant reconstruction, while 20 underwent reconstruction with a tissue expander. Concerning chemo-/radiotherapy, 27 patients received this treatment (Table 4).

The results of the SWMT at the preoperative and postoperative intervals are shown in Table 5 as mean values for each of the nine predefined points evaluated.

**Figure 2:**
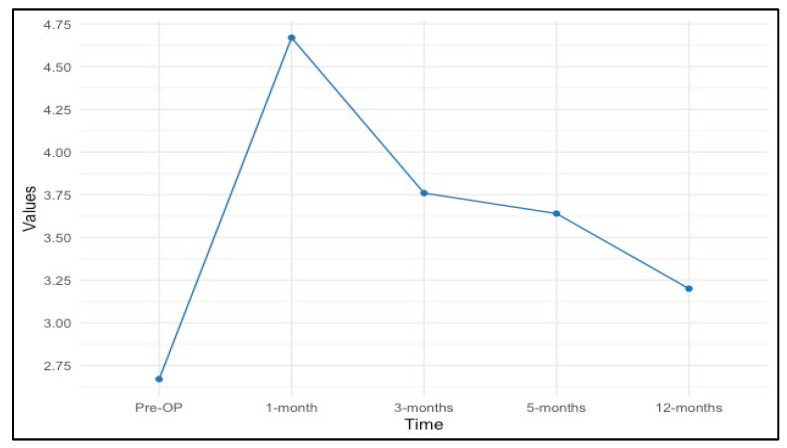
Trend of average sensation values over time.

**Figure 3:**
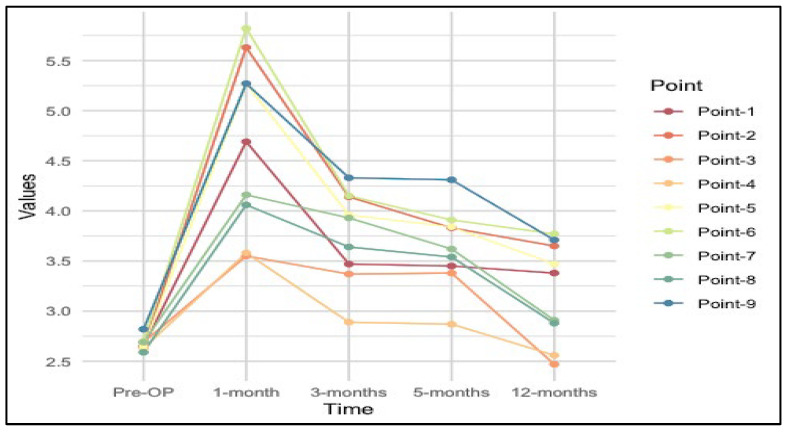
Trend of average sensation values for each predefined point over time.

The following values quantify the visual trend we observed, with an increase in monofilament size values at the 1-month evaluation, followed by a gradual decrease over subsequent months (Figure 2 and Figure 3).

Larger monofilament values correspond to higher pressure/lower sensibility. Therefore, sensation impairment was most prominent at the first postoperative evaluation, followed by improvement throughout the postoperative period.

Preoperative sensation values were substantially consistent across the nine points assessed, ranging approximately from 2.59 to 2.82, corresponding to normal sensation values. There was a noticeable increase in the mean sensation values across almost all points at the 1-month evaluation, with means ranging from 3.55 to 5.82. This increase in filament size corresponds to a decrease in sensation values, which is consistent with the hypothesis that sensitivity will diminish after surgery due to the surgical impact on nerve function.

Sensation values at 3 months after surgery showed variability, with them having decreased scores compared to the 1-month evaluation, marking the beginning of sensation recovery, while remaining elevated compared to preoperative levels, suggesting continued impact on sensation (2.89–4.33). Mean values at the 6-month assessment show improvement compared to 1 and 3 months postoperatively, indicating continued recovery, while remaining inferior to preoperative levels (2.87–4.31). The last postoperative assessment was performed at the 12-month mark, where sensation values showed a trend toward improvement (2.47–3.77).

Using a paired-sample *t*-test, we compared the obtained results, using the preoperative measurements as the control group, to observe which areas of the breast skin and NAC had the best-preserved sensibility. Point 3 stands out as having the best sensation both preoperatively and one year postoperatively, suggesting that this area might have retained or regained its sensation comparatively well. At 1 and 3 months postoperatively, the sensation values were higher, indicating decreased sensation, but Point 3 (at the 1-month evaluation) and Point 4 (at the 3-month evaluation) showed relatively better sensation values than the other areas during these periods. By 6 months, point 4 showed the best sensation recovery, indicating variability in how sensations recovered across different points on the breast. Points 3 and 4 (medial quadrants of the breast) consistently appeared among the top-ranked points across most time points, suggesting that these areas tend to have the most favorable sensation recovery or maintenance over time. Conversely, points 6 and 9 (lower-lateral areola and nipple, respectively) consistently appeared as the lowest-ranked point, indicating that this was the area most affected in terms of sensation loss or slowest recovery. These results may be related to the location of the incision site, in most patients an infero-lateral incision approach was chosen, which would explain the negative effect on these areas’ sensation function. Furthermore, damage to the lateral cutaneous branch (LCB) of the fourth intercostal nerve during surgery, which is the most prevalent contributor of sensation to the NAC, may also explain the reduced sensory results in the nipple. Furthermore, delayed or immediate reconstruction, incision sites, axillary surgery, implant or expander, chemotherapy, and age were evaluated to check for statistically significant effects (*p*-values > 0.05), indicating no statistically significant differences in averaged sensation values due to these factors. This suggests that the timing of reconstruction, the incision site used, whether axillary surgery was performed, the choice between an implant or expander if the patient underwent chemotherapy and the patient’s age do not significantly impact sensation over the entire post-operative period analyzed (Table 6).

The lack of significant impact from other surgical and demographic factors suggests that individual variations in sensation recovery may be influenced by other unexamined factors or the inherent variability in patient responses that should be further analyzed in a larger patient sample for more accurate results. Additionally, it is worth noting that statistical significance does not necessarily equate to clinical significance. Therefore, these findings should be interpreted within the broader context of patient care and other research findings.

### 3.2. Two-Point Discrimination Test

The two-point discrimination test was conducted in the four quadrants of the breast. The results described whether the patient was able to perceive two points, only one point, or no point at all. Further interpretation of the results was defined as fair to normal sensation if the patients perceived 2 points, protective sensation if one point is perceived, and anesthesia when no point is perceived. Variability was observed in the results of the two-point discrimination test, indicating a possible influence of surgery and recovery time. The results for the TPD test are shown as percentages in Table 7 and Table 8, where 1 represents the upper lateral quadrant, 2 represents the lower lateral quadrant, 3 represents the lower medial quadrant, and 4 represents the upper medial quadrant.

A significant proportion of patients (59.58%) exhibited fair to normal sensation in most quadrants, with 40.52% showing protective sensation at the pre-operative evaluation. There was a noticeable increase in patients with protective sensation (45.31%) and anesthesia (21.88%), with a decrease in fair to normal sensation (32.81%) at the 1-month post-OP sensation. At 3 months, anesthesia decreased (6.94%), and fair to normal sensation rose again (58.33%).

Over the 3, 6, and 12 months of post-operative evaluations, there was a gradual shift back toward more patients experiencing fair to normal sensation, suggesting sensory function recovery. At the final evaluation, the percentage of patients with fair to normal sensation increased to 53.12%, indicating recovery, but not entirely to preoperative levels. Points 3 and 4 (medial quadrants of the breast) consistently ranked among the top in most time frames, indicating that they generally have the highest proportion of fair to normal sensation compared to other points. Points 2 and 1 (lateral quadrants of the breast) fluctuate in their rankings, not showing as effective maintenance or improvement in sensation throughout time as points 3 and 4. Notably, point 2, which represents the outer lower quadrant of the breast, reflects the lowest two-point discrimination results. These results may be related to the location of the incision site, in most patients an infero-lateral incision approach was chosen, which would explain the negative effect on this quadrant’s sensation function. The results further indicate point 4’s high ranking across multiple time points and affirm it as the point with generally better sensation recovery or maintenance, as it most frequently exhibits a high score.

### 3.3. Assessment of the Patients’ Quality of Life

The patients’ quality of life was measured using the BREAST-Q questionnaire, with sections filled in before and after surgery. Three domains were evaluated: overall satisfaction, quality of life, and satisfaction with reconstruction results. The following were the Rasch Transformed (RT) scores obtained from the BREAST-Q, scored from 0–100, where 0 indicates the worst and 100 corresponds to the best results. Higher scores reflect a better outcome (Table 9).

The results of the BREAST-Q suggest a trend towards improvement in the patient’s quality of life, indicating post-surgical adaptation and recovery, and significant satisfaction with reconstruction. Out of the 42 patients evaluated in the study, 4 did not complete the BREAST-Q, resulting in a total of 38 completed questionnaires. Patients reported a moderate level of satisfaction with breasts pre-operatively (53%), which served as a baseline for comparing post-operative satisfaction results. Satisfaction with breast reconstruction shows a very slight decrease in satisfaction (51%), indicating that patients maintain a high level of satisfaction score, which, while being moderately decreased compared to preoperative satisfaction, still reflects a positive outcome. The proximity of these values suggests that the surgical results were well received.

The scores for satisfaction with implant and nipple reconstruction are particularly noteworthy, with patients expressing substantial contentment, scoring 5 out of 8 and 3 out of 4, respectively. Quality of Life results show generally favorable results, with Psychosocial Well-Being being rated at 64%. Sexual Well-Being and Physical Well-Being Chest have lower scores at 50% and 36%, respectively. The lowest score was for Adverse Effects of Radiation (29%), indicating a significant negative impact due to this therapy’s adverse effects.

Overall, the BREAST-Q scores suggest that patients experience a high level of satisfaction with their reconstructive outcomes.

### 3.4. Limitations of the Study

It is important to recognize the limitations of this study. These include our relatively small sample size, as well as the fact that it was conducted as a single-center design. Additionally, there are potential confounding factors such as age, comorbidities, and variations in surgical techniques, which may impact the generalizability of the results and limit their applicability to broader populations.

To address these limitations, future research efforts should include multicenter studies with a larger and more diverse patient pool, using standardized assessment methods, and incorporating other objective measures, such as neuroimaging techniques.

## 4. Discussion

### 4.1. Nipple-Sparing Mastectomy and Breast Reconstruction

Breast cancer is a pervasive malignancy among women that requires surgical management as the primary treatment option. Individual patient preferences play a significant role in the selection of treatment methods and management strategies, including surgical techniques. NSM with implant-based reconstruction has become a preferred approach by surgeons and patients, on account of good aesthetic outcomes, low complication rates, and reduced adverse psychological impacts of mastectomy [7,8,9]. Surgeons are searching for surgical techniques that provide the best aesthetic results and patient QoL outcomes. Several studies reflect on how mastectomies, where the NAC is preserved, reveal better QoL results post-operatively, including better body image and sexual functioning, when compared with non-nipple-sparing mastectomies [10].

During the Oncoplastic Breast Consortium conference on NSM, most doctors felt that the oncological safety of NSM was similar to that of conventional mastectomy without reconstruction or breast-conserving surgery. However, there was unanimous agreement that clinical evidence of nipple involvement, bloody nipple discharge, inflammatory breast cancer, and any R1 resection at the nipple margin are oncological contraindications to nipple preservation [7,11].

According to a recent analysis from the Surveillance, Epidemiology, and End Results (SEER) database, the 5- and 10-year cancer-specific survival and overall survival rates, in breast cancer patients who underwent NSM from 1998 to 2013, were 96.9%, 94.9%, 94.1%, and 88.0%, respectively [12]. Building on the safety of this procedure, Metere et al. [13]. reported that the principal early complications of NSM were necrosis of the NAC (6.4%), depigmentation (3.1%), and nipple necrosis (2.8%). Most patients presented with no early or late complications (83.2% and 65.8%, respectively). Moreover, the local relapse rates at the NAC level and in the skin were 4.9%.

Both of these studies provide evidence supporting the safety of NSM, with survival and relapse rates comparable to those of more invasive surgical treatments, including skin-sparing mastectomy and modified radical mastectomy.

Breast reconstruction is a vital stage in the recovery process of patients who have undergone NSM, in which the breast is rebuilt with the goal of creating a more natural breast appearance and restoring positive body image perception. Immediate breast reconstruction results in better cosmetic outcomes, shorter costs, quicker recovery, higher quality of life, and increased psychological well-being. Delayed reconstruction is a beneficial option for post-mastectomy radiation therapy (PMRT) cases and reduces the incidence of postoperative complications [14]. Furthermore, recent research indicates that pre-pectoral implant-based reconstruction following neoadjuvant chemotherapy results in improved aesthetic outcomes and quality of life, suggesting its effectiveness as a treatment option [15]. In the current literature, there is an ongoing debate regarding direct-to-implant (DTI) and expander-based (EB) techniques, discussing their complications, differences, and outcomes in breast reconstruction. A study by Riggio et al. [16] with a total of 120 patients (45% with EB and 55% with DTI) focused on patient satisfaction and well-being between these two techniques. The findings of this study demonstrated that these results did not differ between EB and DTI in patients following NSM.

Understanding the distinguishing characteristics and outcomes of these breast reconstruction techniques is essential for the creation of tailored treatments, specific to the patients’ individual preferences, to maximize patient satisfaction and well-being.

### 4.2. Innervation of the Breast and Nipple-Areolar Complex and Sensory Changes in Patients Undergoing Breast Surgery

The neuroanatomy of the breast encompasses a network of nerves involved in supplying sensory functions to the breast skin and NAC. This network plays a crucial role in sensory perception and its main contributors are branches of the intercostal nerves T2–T6, particularly the anterior and lateral cutaneous nerves [17]. While the anterior cutaneous branches (ACBs) and lateral cutaneous branches (LCBs) of the second to sixth intercostal nerves innervate the breast skin, the NAC is primarily innervated by the ACBs and LCBs of the third to fifth intercostal nerves. Notably, a greater part of the breast skin surface and NAC are supplied by the ACB and LCB of the fourth intercostal nerve. The most consistent contributory nerve to the NAC is the LCB of the fourth intercostal nerve [18].

Beyond these primary suppliers of the breast innervation network, the neural network of the breast can be further described by areas of the breast based on the current literature. The supraclavicular branches of the cervical plexus play a minor role in the sensory physiology of the upper breast. The lateral and medial regions of the breast are innervated by the lateral and anterior cutaneous branches, respectively. The sensory function of the lower part of the breast is provided by smaller branches derived from the sixth intercostal nerve [19,20]. Furthermore, tactile sensitivity shows variability when comparing different areas of the breast, with the highest being the nipple, followed by the upper quadrant of the breast, areola, and lower quadrant of the breast [20].

Overall, while breast sensitivity is mainly contingent on the anterior and lateral cutaneous nerves, it is important to note the complexity and anatomical positioning of the neural network of the breast to avoid damaging these nerves during surgery to preserve breast and NAC sensation.

In addition to good aesthetic results and breast appearance, the integrity of breast physiology is becoming an important goal of breast surgery. Breast skin and NAC sensitivity recovery have a significant impact on patients’ QoL after surgery. Therefore, it is fundamental to have sensory function recuperation as an objective. For instance, surgeons should avoid damaging the anterior and lateral cutaneous branches of the third, fourth, and fifth intercostal nerves during NSM or reconstructive breast procedures. Particular attention should be directed to preventing the compromise of the fourth intercostal nerve, which is the primary sensory innervation source of the NAC [18,21].

Bijkerk et al. [22]. demonstrated that there is a statistically significant impairment of cutaneous threshold sensibility to touch after mastectomy as compared to a healthy, non-operated breast, notwithstanding the time since the surgery.

A recent study on sensory sensitivity alterations in breast surgery addressed the influence of the surgical incision position on postoperative sensory recovery. Regarding NSM, reduced NAC sensitivity was observed after surgery using areolar and submammary incisions, potentially because of damage to the cutaneous branch of the fourth intercostal nerve. During NSM, a small and short incision contributes to better postoperative sensitivity [20]. Special consideration should be given to the orientation of the cutaneous sensory nerves at the incision site, as well as incision positioning and size, to reduce sensory abnormalities. Additionally, they mentioned that sensitivity recovers more quickly in the nipple than in the areola and upper quadrant than in the lower quadrant.

### 4.3. Peripheral Nerve Regeneration

The peripheral nervous system can self-repair following injury. The regeneration period correlates with the synthesis and subsequent transportation of intracellular substances. Thus, peripheral nerve regeneration is often slow. Axonal sprouts extend at a rate of approximately 1–3 mm/day [23,24].

Two main pathways can lead to reinnervation. This can occur through collateral branching of non-injured axons or regeneration of damaged axons. Collateral branching is the main mechanism of recovery when 20–30% of axons are injured. However, if a lesser collection of axons is damaged, collateral sprouting is the primary process. This entails intact motor axons generating sprouts for reinnervation of the denervated muscle. As early as four days after injury, these sprouts develop from the nodes of Ranvier (nodal sprouts) or nerve terminals (terminal sprouts). Collateral sprouting will allow for clinical recovery spanning from 3–6 months from the time of injury [24,25]. On the contrary, in the presence of a severe or complete injury, nerve regeneration will only begin following Wallerian degeneration, with axon regeneration being the principal mechanism towards clinical recovery from 6 to 24 months succeeding injury [24].

Unintentional peripheral nerve damage during surgery may lead to sensory abnormalities. Considering the chapter on peripheral nerve trauma from the Neurology in Clinical Practice Book and its description of nerve regeneration periods, we can expect that our sensibility assessment results should reflect these time frames.

The interpretation of our results using the manual guide for baseline tactile Semmes-Weinstein monofilaments reflects previously described nerve regeneration times, as it shows us how the patients had normal sensation prior to the operation, loss of protective sensation at the 1-month evaluation, and diminished protective sensation values at the 3-month evaluation, with an improvement in diminished light touch values at the 6- and 12-month evaluations.

The findings of our study provide a comprehensive view of sensitivity recovery after NSM followed by reconstruction with an implant or expander while shedding light on the positive outcomes associated with this procedure in terms of the breast skin and NAC sensation and its impact on the patient’s QoL. Focusing on long-term postoperative sensation restoration to the breast skin and NAC, which is a crucial aspect frequently compromised during NSM, our results provide a clear analysis of the central tendency and evolution of sensibility values throughout the postoperative process.

Consistent with the previous literature, our results reflect a steep decrease in sensation values at the 1-month sensation assessment when compared with the pre-operative values [26]. This decline is indicative of the expected acute sensory impairment associated with surgical trauma, tissue manipulation, and nerve disruption during NSM followed by reconstruction. However, we observed a notable trend of gradual sensory recovery over the subsequent evaluations up to 1-year, pointing to a capacity for nerve regeneration, tissue remodeling, and sensory reintegration over time. Additionally, our results provide insight into how sensation recovery varies across different areas of the breast postoperatively, with some areas showing better recovery or maintenance of sensation than others do.

The underlying mechanisms that potentiate this sensory recovery should be further investigated, including the role of neuroplasticity, collateral nerve sprouting, and vascularization in promoting sensory restoration in reconstructed breasts. Moreover, a study by Tevlin et al. suggested the possibility of targeted nipple-areolar complex re-innervation as a method to enhance recovery of NAC sensation post reconstruction [27]. While our study did not employ such techniques, these findings highlight an area for potential surgical innovation in NSM procedures.

The evolution of surgical techniques, as noted by Wang et al., also plays a crucial role in improving patient outcomes [28]. Our study’s technique maintained a conservative approach to NAC preservation, which may have contributed to the observed trends in sensation recovery. Further research into surgical techniques, particularly those that preserve nerve integrity, could prove beneficial.

When contextualizing our findings with the existing literature, we observed that the improvement in tactile pressure sensitivity follows a similar trend to that described in previous studies. The variation in sensory recovery between breast quadrants, as evidenced by the TPD test, indicating a possible influence of surgery and recovery time, has significant clinical implications.

The obtained scores from the SWMT and the TPD test reflect better sensation recovery or maintenance in the medial quadrants of the breast compared to the lateral quadrants of the breast, throughout the postoperative period. Our results suggest that attention to sensitivity in specific areas may be crucial to optimizing postoperative quality of life.

### 4.4. Quality of Life Evaluation

The BREAST-Q is a patient-reported outcome measure questionnaire that provides insights into the impact and outcomes of breast cancer surgery and reconstruction from the patient’s perspective. The results obtained from this questionnaire have the capability to support advocacy, quality metrics, and an evidence-based approach to surgical practice by quantifying and highlighting key factors that contribute to health-related QoL and patient satisfaction [29,30].

A variety of medical papers have analyzed how different mastectomy techniques, reconstructive surgery, and sensation loss impact patients’ QoL using BREAST-Q. These reflect on the positive effect of NSM followed by reconstruction on patients’ psychosocial and sexual well-being when compared with conventional skin-sparing mastectomy and nipple reconstruction techniques, as well as in patients who have undergone total mastectomy [31,32]. Furthermore, post-mastectomy reconstruction also has a positive impact on the sexual and psychosocial well-being of patients. For instance, a study by Vohra et al. concluded that patients who underwent total mastectomy with reconstruction had higher sexual and psychosocial health scores than those who underwent only total mastectomy. However, the most satisfied with their cosmetic outcome were the patients who received breast-conserving surgery, followed by total mastectomy with reconstruction and without reconstruction [33].

Moreover, Valdivia et al. also came to the conclusion that the group with reconstruction reflected better results in satisfaction and QoL than patients without reconstruction, as they presented significant differences in the breast satisfaction and sexual well-being scales [34].

Equally noteworthy are the results of the BREAST-Q, patient-reported outcome measures questionnaire, which allow us to assess the implications of this procedure on the patient’s overall QoL, from the patient’s perspective. Overall, the BREAST-Q scores suggest that patients experience a high level of satisfaction with their reconstructive outcomes. In conclusion, to optimize patients’ QoL during the postoperative period, it is of paramount importance to devise a thorough surgical and clinical care plan. Taking into consideration different mastectomy techniques, reconstructive surgery, and sensation restoration of the breast and NAC, it is possible to improve patients’ sexual health, psychological well-being, and overall satisfaction.

## 5. Conclusions

This study confirms that sensibility diminishes after NSM followed by reconstruction, as observed when comparing the sensation evaluation results before the operation with the 1-month evaluation, with a small progressive return reflected in the following months of the postoperative period. Furthermore, the BREAST-Q scores reflect a high level of patient satisfaction with reconstructive outcomes following this procedure. The conclusions obtained from the sensibility evaluation tests combined with the BREAST-Q will contribute further insights and evidence to the medical community, potentially facilitating the improvement of the observed results. The implications of our findings are significant, as they highlight the importance of considering these sensations when planning and performing nipple-sparing mastectomy procedures with reconstruction. We plan to build on these insights and continue to explore the impact of preserving breast skin and NAC sensation. Identifying sensory changes in diverse settings will enhance the current results in the field of breast oncology and reconstruction. In addition, it will ensure suitable postoperative support and care with the long-term objective of providing the best possible QoL results for patients.

## Figures and Tables

**Table 1:** Nine predefined points for sensation evaluation of the breast skin and NAC.

1	2	3	4	5	6	7	8	9
Upper- Lateral Quadrant (ULQ)	Lower- Lateral Quadrant (LLQ)	Lower- Medial Quadrant (LMQ)	Upper- Medial Quadrant (LMQ)	Upper- Lateral Areola (ULA)	Lower- Lateral Areola (LLA)	Lower- Medial Areola (LMA)	Upper- Medial Areola (UMA)	Nipple

**Table 2:** Semmes-Weinstein monofilament test values and corresponding interpretation.

Grades	Monofilament Size	Target Force (gm)	Interpretation
5	1.65–2.83	0.008–0.07	Normal
4	3.22–3.61	0.16–0.4	Diminished light touch
3	3.84–4.31	0.6–2	Diminished protective sensation
2	4.56–4.93	4–8	Loss of protective sensation
1	5.05–6.45	10–180	Loss of protective sensation
0	6.65	300	Loss of sensation/Deep pressure sensation only

Manual: Baseline^®^ Tactile™ Semmes-Weinstein type monofilaments. Fabrication Enterprises Inc. Authorized CE representative: RMS UK Ltd. 28 Trinity Road. Nailsea, Somerset BS48 4NU (UK). ©2017, all rights reserved. Baseline and Tactile are trademarks of Goldberg.

**Table 3:** Two-point discrimination test values and corresponding interpretation.

Measurement	Interpretation
2 mm to 5 mm	Normal
6 mm to 10 mm	Fair
11 mm to 15 mm	Poor
One point of perception	Protective
No point perceived	Anesthesia

American Society for Surgery of the Hand. *The hand, examination and diagnosis.* New York: Churchill Livingstone; 1990.

**Table 4:** Patient characteristics.

	Implant	Expander	Nº Patients	Nº Breasts
Bilateral	12	12	24	48
Unilateral	10	8	18	18
Total	22	20	42	66

**Table 5:** Tactile pressure sensitivity.

Time	Pre-OP	1-Month	3-Months	6-Months	12-Months
Point-1	2.65	4.69	3.47	3.45	3.38
Point-2	2.65	5.63	4.14	3.83	3.65
Point-3	2.69	3.55	3.37	3.38	2.47
Point-4	2.63	3.58	2.89	2.87	2.56
Point-5	2.65	5.27	3.96	3.84	3.47
Point-6	2.70	5.82	4.15	3.91	3.77
Point-7	2.69	4.16	3.93	3.62	2.91
Point-8	2.59	4.06	3.64	3.54	2.88
Point-9	2.82	5.27	4.33	4.31	3.71
Total Breast	2.67	4.67	3.76	3.64	3.20
*p*-value	<0.001	<0.001	<0.001	<0.001	<0.001

**Table 6:** Parameters.

Parameter	*p*-Value
Immediate or Delayed Reconstruction	0.267
Incision site	0.421
Axillary Surgery	0.476
Chemotherapy	0.535
Implant or Expander	0.439
Side	0.577
Age Correlation (Spearman)	0.561

**Table 7:** Two-point discrimination test results presented as percentages.

Points Perceived	Pre-OP	1-Month	3-Months	6-Months	12-Months
0 points	N/A	21.88%	6.94%	8.33%	6.25%
1 point	40.52%	45.31%	34.72%	52.38%	40.62%
2 points	59.48%	32.81%	58.33%	39.29%	53.12%

**Table 8:** Two-point discrimination test results presented as percentages for each quadrant.

Time Point	Quadrant	Anesthesia (0)	Protective Sensation (1)	Fair/Normal Sensation (2)
Preoperative	1	N/A	48.28%	51.72%
	2	N/A	37.93%	62.07%
	3	N/A	44.83%	55.17%
	4	N/A	31.03%	68.97%
1 month	1	18.75%	56.25%	25.00%
	2	62.50%	31.25%	6.25%
	3	6.25%	50.00%	43.75%
	4	N/A	43.75%	56.25%
3 months	1	11.11%	38.89%	50.00%
	2	11.11%	38.89%	50.00%
	3	5.56%	33.33%	61.11%
	4	N/A	27.78%	72.22%
6 months	1	9.52%	61.90%	28.57%
	2	19.05%	47.62%	33.33%
	3	4.76%	66.67%	28.57%
	4	N/A	33.33%	66.67%
12 months	1	N/A	37.50%	62.50%
	2	12.50%	50.00%	37.50%
	3	12.50%	43.75%	43.75%
	4	N/A	31.25%	68.75%

**Table 9:** Breast questionnaire results.

Domains	BREAST-Q Scale	RT Score (%)
Overall Satisfaction	Satisfaction with Breast Pre-OP	53
	Satisfaction with Breast Reconstruction	51
	Satisfaction with Implant	5 (max 8Pts)
	Satisfaction with Nipple Reconstruction	3 (max 4Pts)
Quality of Life	Psychosocial Well-Being	64
	Sexual Well-Being	50
	Physical Well-Being Chest	36
	Adverse Effects of Radiation	29

## Data Availability

The data presented in this study are available on request from the corresponding author. The data are not publicly available due to ethical and privacy reasons.
